# Uncompromised MRI of knee cartilage while incorporating sensitive sodium MRI

**DOI:** 10.1002/nbm.4173

**Published:** 2019-09-10

**Authors:** S. Brinkhof, A. Ali Haghnejad, K. Ito, K. Markenroth Bloch, D.W.J. Klomp

**Affiliations:** ^1^ Department of Radiology University Medical Center Utrecht Utrecht Netherlands; ^2^ Wavetronica Netherlands; ^3^ Department of Orthopaedics, University Medical Center Utrecht, Utrecht, Netherlands; ^4^ Orthopaedic Biomechanics, Department of Biomedical Engineering Eindhoven University of Technology Eindhoven Netherlands; ^5^ Lund University BioImaging Center Lund Sweden

**Keywords:** 7 T, cartilage, knee, sodium

## Abstract

Sodium imaging is able to assess changes in ion content, linked to glycosaminoglycan content, which is important to guide orthopeadic procedures such as articular cartilage repair. Sodium imaging is ideally performed using double tuned RF coils, to combine high resolution morphological imaging with biochemical information from sodium imaging to assess ion content. The proton image quality of such coils is often harshly degraded, with up to 50% of SNR or severe acceleration loss as compared to single tuned coils. Reasons are that the number of proton receive channels often severely reduced and double tuning will degrade the intrinsic sensitivity of the RF coil on at least one of the nuclei. However, the aim of this work was to implement a double‐tuned sodium/proton knee coil setup without deterioration of the proton signal whilst being able to achieve acquisition of high SNR sodium images.

A double‐tuned knee coil was constructed as a shielded birdcage optimized for sodium and compromised for proton. To exclude any compromise, the proton part of the birdcage is used for transmit only and interfaced to RF amplifiers that can fully mitigate the reduced efficiency. In addition, a 15 channel single tuned proton receiver coil was embedded within the double‐resonant birdcage to maintain optimal SNR and acceleration for proton imaging. To validate the efficiency of our coil, the designed coil was compared with the state‐of‐the‐art single‐tuned alternative at 7 T. B1+ corrected SNR maps were used to compare both coils on proton performance and g‐factor maps were used to compare both coils on acceleration possibilities. The newly constructed double‐tuned coil was shown to have comparable proton quality and acceleration possibilities to the single‐tuned alternative while also being able to acquire high SNR sodium images.

## Graphical abstract

Sodium imaging is able to assess and evaluate changes in glycosaminoglycan content in articular cartilage. Sodium imaging is often performed with double tuned sodium/proton RF coils, of which the proton imaging quality is severely degraded. In this work, a double tuned sodium/proton knee coil setup was implemented with comparable proton image quality to the state‐of‐the‐art single tuned alternative, while also being able to acquire high SNR sodium images.

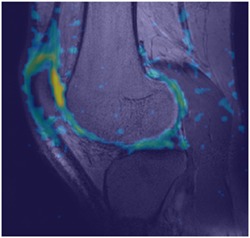

**Abbreviations**
FCD: fixed charge density
GAG: glycosaminoglycans
gagCEST: glycosaminoglycan Chemical Exchange Saturation Transfer
SNR: signal‐to‐noise ratio

## 1 INTRODUCTION

Osteoarthritis (OA) is a degenerative whole‐joint disease which is a large burden on society. Patients with OA suffer from joint stiffness, reduced range of motion of the joints and often pain. The cause of these symptoms is cartilage degeneration, characterized by loss of glycosaminoglycans (GAG) and alterations to the collagen fiber network. The loss of GAG content leads to softening of articular cartilage and therefore a reduced ability to withstand loading and wear of the joint.^1^, ^2^ Early detection of cartilage damage is vital for treatment planning to prevent or decelerate irreversible damage to the joint. The treatment planning is often hampered by insufficient information from standard‐of‐care MRI, because the lesion size can be underestimated by 60 to 70%.^3^, ^4^ Assessment of early cartilage damage by biochemical MRI can improve this treatment planning. Current MRI methods can quantify the morphology of the joint, but biochemical changes in the joint often occur before morphological changes appear. This gives rise to the need for biochemical and quantitative MRI to capture these intricate changes in the biochemical composition of articular cartilage.

Examples of such biochemical MRI methods include T2 mapping,^5^, ^6^, ^7^ T1ρ mapping,^8^, ^9^ delayed gadolinium enhanced MRI of cartilage (dGEMRIC),^10^, ^11^ glycosaminoglycan chemical exchange saturation transfer (gagCEST)^12^, ^13^, ^14^ and sodium imaging,^15^, ^16^, ^17^ each probing one or more characteristics of articular cartilage. These techniques result in quantitative biomarkers which reflect the biochemical composition of articular cartilage and can assess early cartilage damage. Insight in cartilage damage is vital in diagnosis of cartilage repair, but also as a tool to realize optimal treatment planning. Understanding cartilage biology and damage can also help the follow‐up of treatment of OA patients and be a powerful research tool to assess new treatment paradigms.

One of the signs of early cartilage damage is ion misbalance and its related loss of GAG content, starting on the surface of the cartilage. GagCEST MRI can capture these changes in GAG content at a high resolution, but gagCEST MRI comes with a few drawbacks. These sequences are strongly dependent on a homogenous B0 field, on the exchange rate and successful gagCEST at 7 T is not that easily translated to conventional clinical field strengths like 1.5 T and 3 T since the GAG resonance is close to the water frequency which is difficult to disentangle at these clinical field strengths. The GAG loss can also be quantified by using sodium MRI due to its direct relation with GAG content.^18^, ^19^ Since GAG is negatively charged due to its sulphate and carboxyl groups it will attract positively charged ions, mainly in the form of sodium, to preserve the electroneutrality.^20^ The sodium concentration is proportional to the GAG concentration, because of the fixed charge density (FCD).^21^ Thus, sodium imaging is able to assess the GAG content and evaluate changes in the GAG content. Moreover, the FCD is a well‐established (*ex‐vivo*) biomarker for OA so an imaging strategy that links more directly to FCD simplifies translation to clinical use.

However, sodium imaging intrinsically suffers from a low signal‐to‐noise ratio (SNR) because of its lower gyromagnetic ratio.^22^, ^23^ Nonetheless, the sodium concentration is similar to GAG and in contrast to gagCEST provides positive contrast and practically no background signal. Moreover, higher field MR scanners provide larger SNR, and it is therefore appealing to use 7 T MR for sodium imaging. Sodium imaging is ideally employed using double tuned RF coils, but the proton image quality of such coils is often severely compromised, even up to 30% of SNR loss in severe cases.^24^, ^25^, ^26^ Reasons for this is that the number of proton receive channels are often reduced as compared to single tuned coils, and that double tuning will degrade the intrinsic sensitivity of the RF coil. However, when incorporating an array of single tuned ^1^H receive coils inside a volume sodium transceiver, proton imaging performance may remain intact. Integrating a receive array not only facilitates the possibility for B0 shimming and the ability to provide co‐registered anatomical images, it can still facilitate all of the above mentioned MRI techniques potentially without compromises. Additionally, acceleration with the array is warranted to maintain acceptable overall scan time.

Due to the comprised MRI performance, sodium MRI is often not considered for clinical diagnoses. Though, there is a great interest in sodium imaging in research which is moving towards the clinic in various fields, such as orthopaedics,^19^ neurology^27^, ^28^ and breast imaging.^29^ In order to add sodium MRI for clinical use, one has to make sure that the quality of proton MRI is up to par with the best available coils. Therefore, for this study a dual tuned sodium‐proton coil as transceiver for sodium and transmitter for hydrogen with a high‐density proton receive array was designed. The setup was tested for the proton imaging performance in healthy volunteers and compared with a state‐of‐the‐art single tuned knee coil. The aim of this work is to demonstrate that this new double tuned coil configuration besides ^23^Na information also gives proton images with image quality comparable to commercially available single‐tuned coils.

## 2 METHODS

### 2.1 Coil design

**Figure 1:**
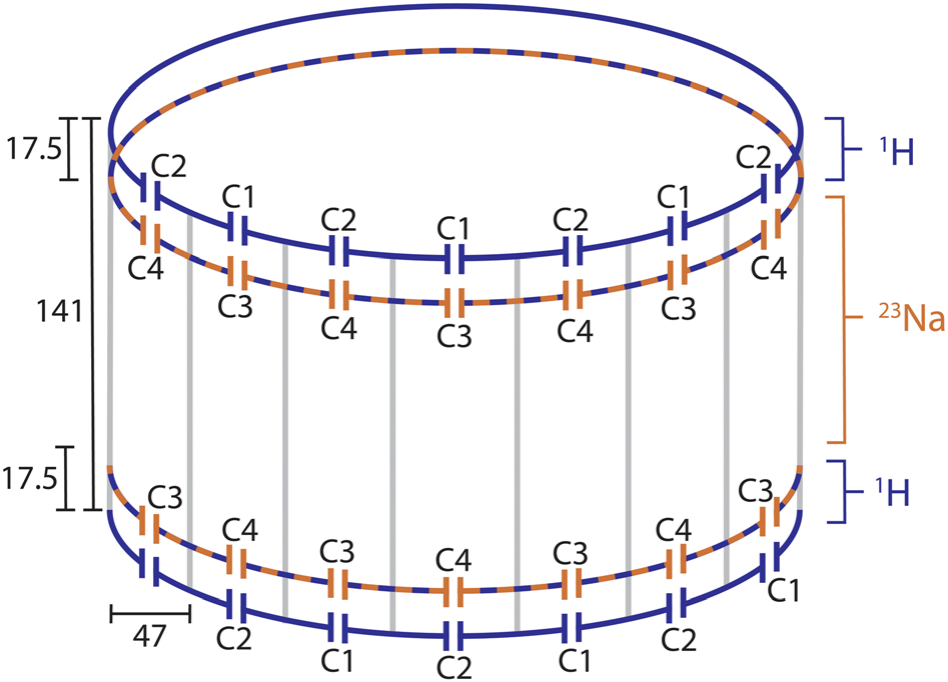
Coil configuration, showing the double‐tuned four‐ring birdcage. The dimensions are in millimeters. The following capacitor values are used: C1 = 5.6 + 2 pF, C2 = 5.6 + 2.4 pF, C3 = 91 pF and C4 = 91 + 8.2 pF

A double‐tuned knee coil was constructed as a double‐resonant four‐ring shielded birdcage (12 rods with a length of 15 cm) and 15 channel proton receiver coil embedded within the double‐resonant birdcage.^30^ The 1H transmit and receive coils could be actively detuned using standard PIN diode circuitry based on activating a parallel resonant inductor over a tuning capacitor. The fifteen overlapping proton receive elements were each 5 cm in width and 9.5 cm in length and connected to low impedance preamplifiers used for preamplifier decoupling and signal amplification before interfacing to the 7 T MRI system. The double tuned coil is optimized for the sodium frequency by using a tank circuit tuned for tuning the ^1^H frequency and maintaining the natural inductance in the endring as observed for the impedance at the ^23^Na frequency. Due to the intrinsically high impedance of the ^1^H coil on the ^23^Na frequency no sodium trap is inserted on the array. A graphical representation of the coil configuration is shown in Figure 1.

To validate the efficiency of the proton coil, our double tuned coil was compared with a vendor‐built single tuned alternative (birdcage transmit and 28 channel receive; Quality Electrodynamics LLC, Ohio, USA).

### 2.2 MR methods

MRI experiments were carried out on a 7.0 Tesla whole body scanner (Achieva; Philips Healthcare, Best, Netherlands) and all experiments were performed with the double tuned coil and single tuned coil in separate imaging sessions. SNR maps were carried out by making a dynamic noise scan where the first dynamic is used to acquire the image, while the second dynamic is used to acquire noise only with the same scan parameters (no RF or gradients are used for the second dynamic, while signal combinations from coil elements in reconstruction is copied from the first dynamic). The following parameters were used: transversal, multi‐slice fast field echo (FFE) with a Cartesian readout; TE = 1.95; TR = 199; flip angle = 10 degrees; field of view (FOV), 200 x 200 x 100 mm^3^; voxel size, 2 x 2 x 2 mm^3^. The B1+ map was acquired using a dual TR protocol with the following parameters: transversal, multi‐slice FFE with a Cartesian readout; TE = 1.0; TR = 40; TR extension = 160; flip angle = 50 degrees; FOV, 170 x 170 x 100 mm^3^; voxel size, 4 x 4 x 4 mm^3^.

For this purpose, sixteen healthy volunteers were included after explaining the study procedures and informed consent was signed. Eight of the volunteers were examined with the single tuned coil, and the other eight with the double tuned coil. For consistency, the right knee was imaged in all healthy volunteers. In each volunteer, SNR maps and B1+ maps were acquired. In addition, for one volunteer per coil a set of g‐factor maps were calculated. Finally, sodium images were acquired in four of the volunteers scanned with the double tuned coil.

Scan parameters for the sodium protocol were chosen as follows: sagittal, three dimensional FFE with a cartesian readout; TE = 1.61 ms; TR = 100 ms; flip angle = 130 degrees (which was optimized via a flip angle series); FOV, 150 x 150 x 150 mm^3^; voxel size, 3 x 3 x 3 mm^3^; 5 signal averages; total acquisition time of 7 minutes and 45 seconds.

### 2.3 Data analysis

Geometry factor (g‐factor) maps were reconstructed on the scanner using reconstruction software available on the Philips system (delayed reconstruction). A total of nine g‐factor maps was reconstructed, with SENSE accelerations in anterior–posterior (AP) and right–left (RL) to a maximum of three in both directions. The g‐factor maps were reconstructed for both coils to assess differences in acceleration possibilities.

Noise scans were used to reconstruct SNR maps and B1+ maps were created by using a dual TR approach. Noise scans were carried out with same scan and reconstruction (i.e. same weighting between coil elements) parameters as the signal scan, but with RF and gradients off. The noise standard deviation is approximated by 0.8 times the mean noise, since the noise is a Rayleigh distribution.^31^ Dividing the signal by this noise standard deviation results in a SNR map. Considering scans are obtained in the regime that SNR is linearly related to the (low) flip angle, this SNR map is normalized for actual used flip angle using the B1+ maps to translate the SNR maps to sensitivity maps and properly assess differences between the double tuned and single tuned coil. Regions of interest (ROI) were drawn in the trochlear cartilage to compare the SNR performance between both coils.

Sodium signal intensity was converted to sodium concentration making use of a calibration curve. Four phantoms filled with known sodium concentrations (75, 150, 225 and 300mM) were placed on the side of the knee and measured together with the volunteers. These four sodium concentrations were used to create a linear calibration curve, which was consequently used to correct the sodium signal intensity to sodium concentration. The sodium concentration map was corrected for the assumed 75% of water content.^20^

## 3 RESULTS

**Figure 2:**
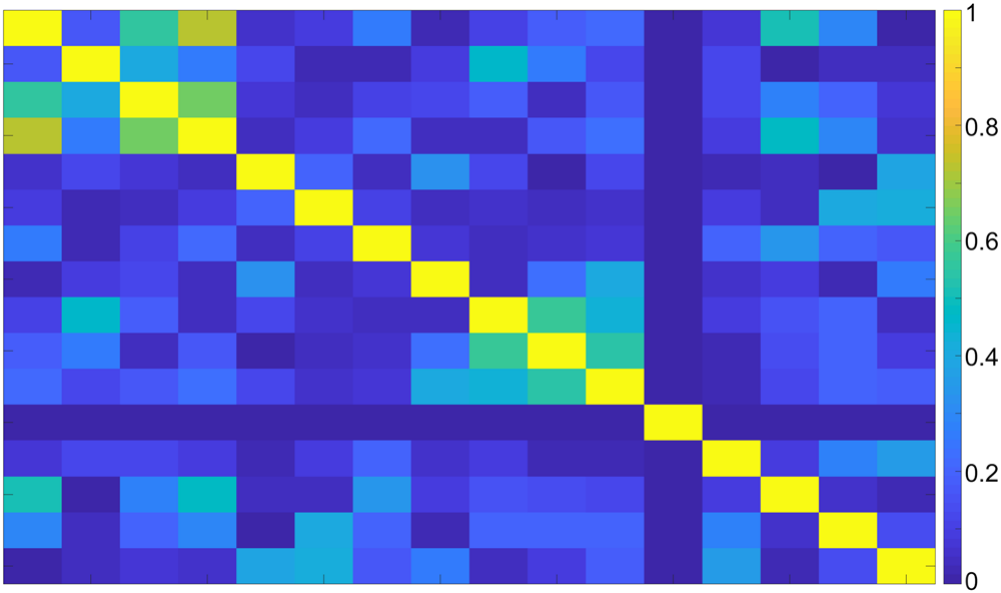
Noise correlation matrix of the double‐tuned coil. A small amount of coupling can be observed between channel 1 and 4, and 1 and 3

**Figure 3:**
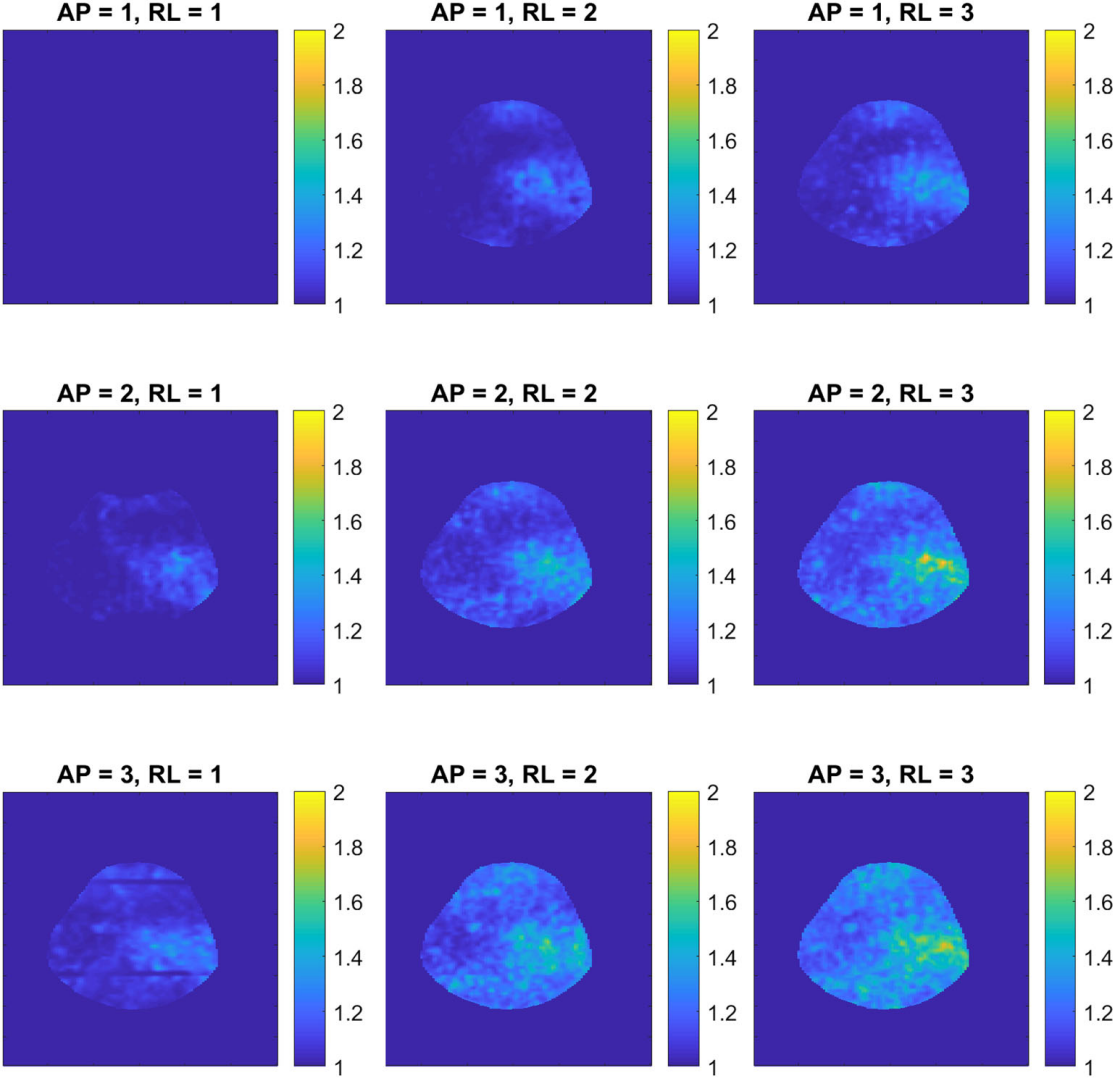
Axial g‐factor maps of double tuned coil, with SENSE acceleration factors up to 3 in both directions

**Figure 4:**
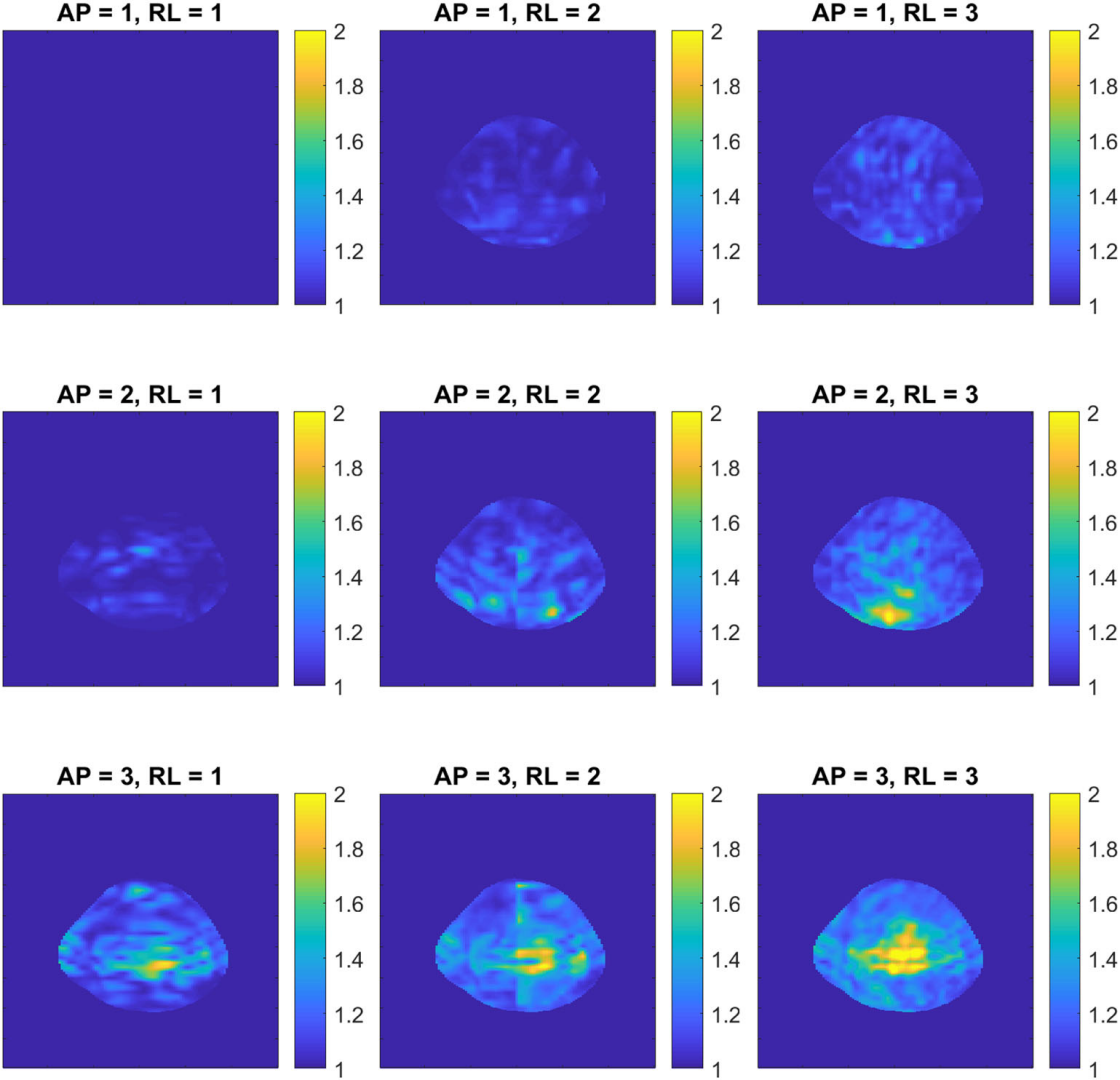
Axial g‐factor maps of the single tuned coil, with SENSE acceleration factors up to 3 in both directions

**Figure 5:**
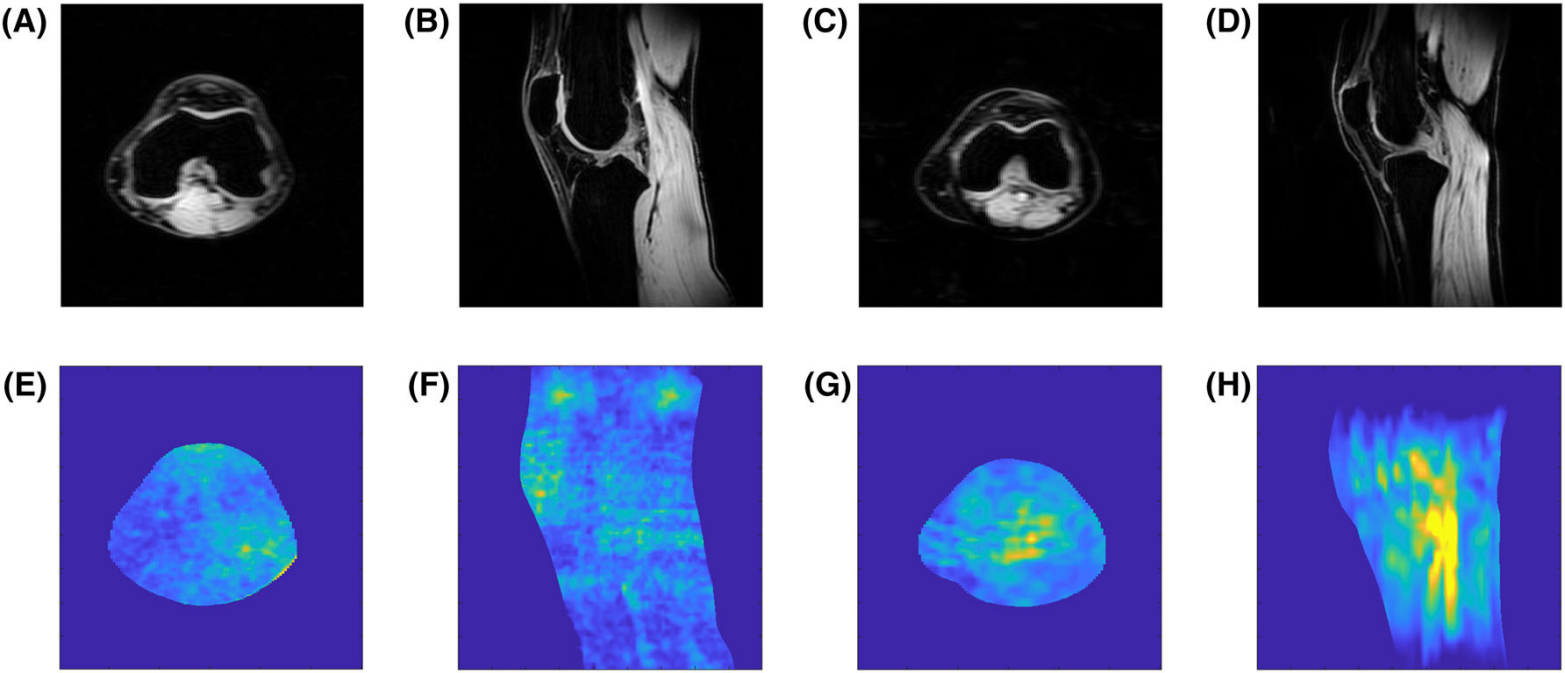
Axial (a) and sagittal (B) image slices for double tuned coil with corresponding g‐factor maps (E & F), and axial (C) and sagittal (D) image slices for single tuned coil with corresponding g‐factor maps (G & H)

**Figure 6:**
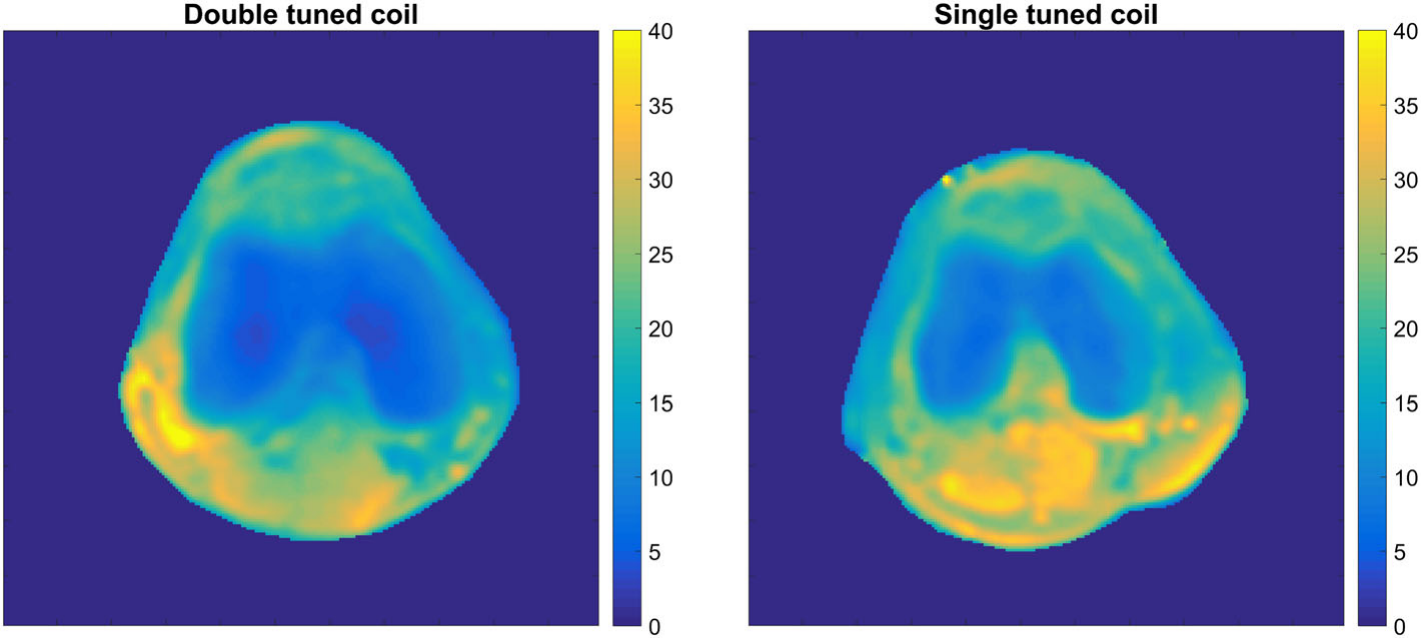
SNR maps of double tuned coil (left) and single tuned coil (right)

Figure 2 shows the noise correlation between the ^1^H receiver elements. Moreover, a low (maximum −19.0 dB, average − 30.7 dB) RF coupling between the ^23^Na transceiver coil and ^1^H receivers was measured at the ^23^Na frequency, which confirms that the ^1^H receivers are invisible for the ^23^Na transceiver. Figure 3 shows the axial g‐factor maps of our double tuned coil, which indicated that a SENSE acceleration of 9 was feasible in this coil when accepting a factor of 2 enhanced noise (AP = 3, RL = 3, maximum g‐factor 1.9). Figure 4 shows the same axial g‐factor maps, acquired with the single tuned coil where the g‐factor maps had a similar hotspot at SENSE acceleration of 9, with a maximum g‐factor of 2.2. Figure 5 shows a typical axial and sagittal slice for each coil with corresponding g‐factor maps (with SENSE AP = 3, RL = 3). The g‐factor maps were acquired in axial slices on the same height as SNR maps in Figure 5, with patella and femur visible on the slice. SNR maps are shown in Figure 6 for our double tuned coil and the single tuned coil, indicating that both had similar SNR. The SNR maps were corrected for B1+ performance, hence reflecting intrinsic coil sensitivity. Table 1 shows the SNR values of both coils for all volunteers. Average SNR in the trochlear cartilage was 29.87 in the double tuned coil (standard deviation = 4.60) and 28.3 in the single tuned coil (standard deviation 1.61). The difference in B1+ efficiency between the two setups was on average 10% in favor of the single tuned coil, which was well within the limits of the RF amplifier (i.e. 2 x 4 kW).

**Table 1:** Overview of SNR values in the trochlear cartilage in both coils

	Double tuned coil		Single tuned coil	
Volunteer	Mean	Standard deviation	Mean	Standard deviation
**1**	32.80	0.68	24.60	0.88
**2**	27.63	1.89	29.03	0.98
**3**	23.04	0.81	27.09	1.73
**4**	33.07	1.61	29.28	2.00
**5**	39.03	1.81	29.73	1.87
**6**	28.34	2.40	29.25	0.76
**7**	28.50	1.40	29.32	1.78
**8**	26.60	1.34	28.24	1.94
**Average**	**29.88**	**4.60**	**28.32**	**1.61**

**Figure 7:**
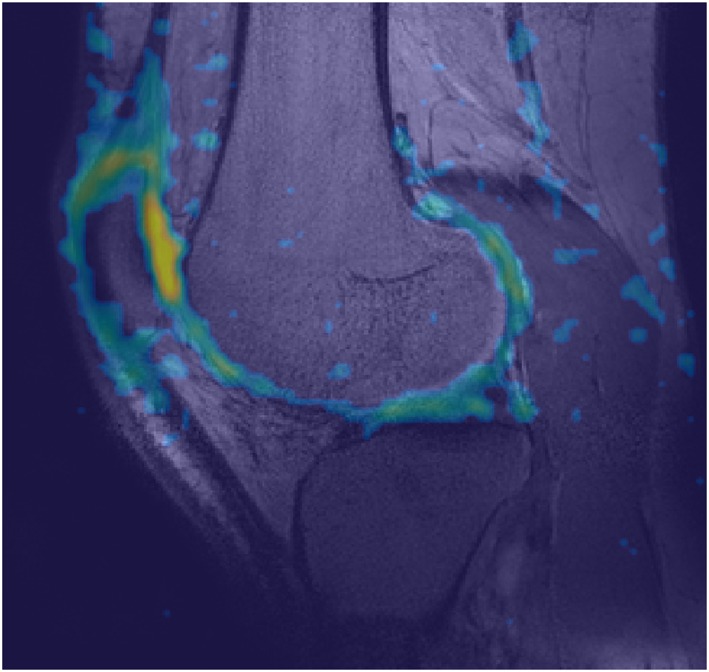
Sodium image overlaid on proton density weighed anatomical scan

Figure 7 shows a sodium scan from the double tuned coil overlaid on a proton density weighed anatomical scan. Cartilage sodium values are in line what has been reported in literature (around 250mM in healthy cartilage tissue).^16^, ^19^

## 4 DISCUSSION

This work presents a double tuned coil optimized for knee imaging with similar SNR and acceleration performance for ^1^H as the state‐of‐the‐art single tuned alternative, because of the combined implementation of local ^1^H receive coils with an optimized volume sodium coil. The double tuned coil can produce proton images with high sensitivity and acceleration possibilities, combined with a high SNR sodium map to assess the glycosaminoglycan content in the articular cartilage in the knee. Acquiring proton scans and sodium scans in the same session, without repositioning the patient, gives high‐resolution anatomical images co‐registered with sodium scans for excellent morphological reference.

The double tuned coil is built as a double‐resonant shielded birdcage with 15 channel proton receive coil embedded within the birdcage. Relatively long receive elements were used within this design to maximize the tissue loss dominance and thereby of the sensitivity of the receive field. This design has no multiple elements in the Z direction, and therefore sacrifices acceleration possibilities in this direction.

This double‐resonant birdcage has a lower efficiency compared to a single‐tuned alternative. Since the diameter is relatively small in comparison to for instance a head coil, there is enough power available to compensate for the reduced efficiency. Moreover it should be noted that the efficiency reduction is only 10% in contrast to the 50% of some earlier work, which can be because the commercial birdcage coil did not needed to be designed for highest efficiency due to the presence of the high sensitivity receiver array. In our setup, the birdcage had to be efficient for it was used for sodium detection as well.

A quadrature sodium transceiver coil is preferred over a setup with multiple elements, because the intrinsic SNR in the center will be similar. A sodium array might be advantageous when higher SNR is needed at the periphery. In addition, any acceleration is not relevant, because T1 of sodium is very short and a fair number of signal averages is already needed to achieve reasonable SNR. Lastly, the T1 of sodium is very short, leading to a high spatial encoding speed. Therefore, a quadrature sodium transceiver coil will be the optimal setup.

To use the proton coil for superb MRI in the same scanning session, one has to be sure that it delivers similar image quality as a state‐of‐the‐art standard single‐tuned proton coil. Within this work, we chose to compare our coil to the state‐of‐the‐art single tuned coil available for 7 T, with the comparison based on the SNR maps and acceleration possibilities. Eight volunteers were included and scanned with the double tuned coil and another eight volunteers scanned with the single tuned coil. Ideally, the same eight volunteers would have been scanned with both coils, but this was logistically impractical. However, the low variation in SNR between individuals (Table 1) indicates that using the same volunteers in both coils would not have altered our results.

The g‐factor maps of the double tuned coil showed the same acceleration possibilities as the single tuned coil. Both coils lost some performance with acceleration factor of 9 (AP = 3, RL=3, g‐factor ~ 2). The patterns within the g‐factor maps showed some differences, which could be attributed to the differences in receive array designs. We opted for long receive elements within our coil, giving a higher tissue load dominance, though, a more diffuse hotspot pattern can be observed. The array in the single tuned coil has smaller loops, which give higher SNR on the surface but lowering the SNR in the middle of the coil. The design of the single tuned coil leads to the typical ‘aliasing’ artefacts in the g‐factor maps as shown in the bottom right in figure 2.

Often, dual tuned coils severely lose SNR on the proton side because optimal sensitivity is desired on the X‐nucleus side, in this case sodium. The sodium transmit efficiency is not compromised, because the coil has been optimized for sodium imaging. The loss of SNR at the proton frequency is often in the range of 20 to 30%,^25^, ^26^ but can account to over 50% of SNR loss in severe cases.^24^ In our work, the SNR maps did not show substantial differences, indicating that the proton performance was not hampered by the addition of a sodium transmit/receive coil. Both coils produce an average B1+ corrected proton SNR of roughly 28 which was shown in eight volunteers per coil. The addition of a sodium transmit/receive coil gives the possibility to acquire high SNR sodium scans in the same session as the proton scans, without changing coils. By implementing an interleaved scanning protocol, sodium and proton scans can be acquired at the same time to be even more time efficient.^32^

Implementing a sodium protocol gives information on the condition of the joint. One of the potential clinical application lies in FCD maps, which can be a proxy for early stage cartilage damage. The sodium concentration map can be converted to the fixed charge density by using the ideal Donnan equilibrium equation.^21^

GAG content can also be visualized by employing a gagCEST imaging protocol, as shown in earlier work.^12^ While the gagCEST signal can be contaminated by signals originating from other compounds or influenced by system imperfections, it can now be obtained in the same scan session as sodium; benefiting from both modalities. The sodium MRI will be more specific but is hindered by lower spatial resolution, and the higher resolution of gagCEST can reveal heterogeneities in more detail.

In conclusion, a double tuned coil was implemented with comparable proton image quality to the state‐of‐the‐art single tuned alternative, while also being able to acquire high SNR sodium images.
